# The kinetics of HTLV-1 proviral load after allogeneic hematopoietic cell transplantation with reduced intensity conditioning regimen; a comparison with that from sibling donors and that from unrelated donors

**DOI:** 10.1186/1742-4690-11-S1-P19

**Published:** 2014-01-07

**Authors:** Ilseung Choi, Ryuji Tanosaki, Atae Utsunomiya, Yoko Suehiro, Jun Okamura, Naokuni Uike

**Affiliations:** 1 Department of Hematology, National Kyushu Cancer Center, Fukuoka, Japan; 2 Clinical Laboratories Division, National Cancer Center Hospital, Tokyo, Japan; 3 Department of Hematology, Imamura Bun-in Hospital, Kagoshima, Japan; 4 Institute for Clinical Research, National Kyushu Cancer Center, Fukuoka, Japan

## 

Adult T-cell leukemia/lymphoma (ATL) is a peripheral T-cell malignancy that is caused by human T-lymphotropic virus type 1 (HTLV-1) with poor prognosis. We have reported the feasibility and efficacy of allogeneic hematopoietic cell transplantation with reduced intensity conditioning (RIC-HCT) for elderly patients with ATL, and have also shown that in more than half of patients the HTLV-1 proviral load (HTLV-PL) decreased to an undetectable level after RIC-SCT, suggested presence of anti HTLV-1 effects. In this study, we try to compare the kinetics of HTLV-PL after RIC-SCT from HTLV-1 antibody negative sibling donor (SD) with that from unrelated donors (UD), all of whom were HTLV-1 antibody negative. Fourteen patients were included in the SD group, and fifteen patients were in UD group. The HTLV-PL decreased to an undetectable revel in 11 of 14 patients (78.6%) in the SD group and 13 of 15 (86.7%) in the UD group with in 120 days after RIC-SCT. In short term, the kinetics of the HTLV-PL after RIC-SCT from UD was almost similar to that from HTLV-1 negative SD. Looking at the long term follow-up results, the HTLV-PL remains to be an undetectable level in only 2 of 11 (18.2%) patients in the SD group (median follow up 111 months [101-122])), on the other hand, 7 of 13 (53.8%) patient in the UD group (24 month [20-36]). RIC-HCT from UD tends to have better anti-HTLV-1 effect, compared with that from SD, but not conclusive because of the short follow-up period.

